# Dissecting the role of toll‐like receptor 7 in pancreatic cancer

**DOI:** 10.1002/cam4.5606

**Published:** 2023-01-05

**Authors:** Maren Stark, Marina Nicolai, Marina Tatura, Corinna U. Keber, Andreas Kaufmann, Ho‐Ryun Chung, Emily P. Slater, Christopher Heeschen, Rita T. Lawlor, Aldo Scarpa, Detlef K. Bartsch, Thomas M. Gress, Stefan Bauer, Malte Buchholz

**Affiliations:** ^1^ Clinic for Gastroenterology, Endocrinology, Metabolism and Infectiology Philipps‐University Marburg Marburg Germany; ^2^ Institute for Immunology, Philipps‐University Marburg Marburg Germany; ^3^ Institute of Pathology, Philipps‐University Marburg Marburg Germany; ^4^ Institute for Medical Bioinformatics and Biostatistics, Philipps‐University Marburg Marburg Germany; ^5^ Department of Visceral, Thoracic and Vascular Surgery Philipps University Marburg Marburg Germany; ^6^ Pancreatic Cancer Heterogeneity Group Candiolo Cancer Institute Candiolo (Torino) Italy; ^7^ ARC‐Net Cancer Research Centre, University of Verona Verona Italy; ^8^ Core facility Small Animal Imaging of the Medical Faculty of the Philipps‐University Marburg Marburg Germany

## Abstract

**Background:**

Toll‐like receptors (TLRs) are gaining attention for their potential to influence tumor biology both on the level of the tumor cells as well as on the level of the surrounding inflammatory stroma. Previous studies resulted in partly conflicting data on the expression of TLR7 in healthy and neoplastic pancreatic tissues as well as its role in pancreatic tumor biology.

**Methods:**

We used qRT‐PCR and immunohistochemistry to asses TLR7 expression in primary patient material and cell lines. Cell viability was analyzed by MTT assay upon incubation with TLR7 agonist/antagonist. Mouse models were used to investigate the role of TLR7 in vivo.

**Results:**

TLR7 is overexpressed in more than 50% of primary human pancreatic ductal adenocarcinoma (PDAC). High TLR7 expression was associated with shorter patient survival, and TLR7 inhibition in cell lines reduced viability in a dose‐dependent manner. In contrast, global TLR7 deficiency did not alter survival or overall histopathological tumor features in genetic mouse models of PDAC.

**Conclusions:**

TLR7 may have opposing functions in tumor versus stroma cells. Further work is required to more precisely dissect the roles of TLR7 and its ligands in different populations of epithelial and stromal cells and to understand their relative contributions to tumor progression.

## Graphical abstract

There are conflicting reports regarding the expression pattern and function of TLR7 in pancreatic cancer. A new study shows that high TLR7 expression correlates with shorter overall survival and its inhibition results in reduced cell viability.

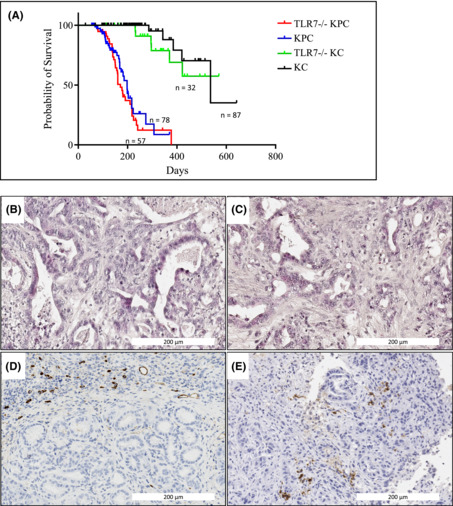

## 1 BACKGROUND

Pancreatic ductal adenocarcinoma (PDAC) exhibits the worst prognosis among all solid tumors with a median survival of 6 months.^1^ Early diagnosis is rare due to the relatively unspecific clinical symptoms, such as back pain, loss of appetite, or weight.^2^ Definite diagnosis is reached by endoscopic ultrasound, computed tomography, or magnetic resonance imaging.^3^ Systemic chemotherapy is currently the only treatment for patients with advanced, metastatic PDAC and therefore new therapies such as efficient immunotherapy are urgently needed. Unfortunately, effective immunotherapy responses in PDAC are rare due to immunosuppressive tumor microenvironment mediated, among others, by myeloid suppressor cells. Therefore, modification of the immune landscape of these tumors by activation or inhibition of innate or adaptive immune pathways may be a promising strategy.

Pattern recognition receptors (PRRs) function in the innate immune system to sense pathogen‐derived molecules and to initiate an appropriate immune response. Nucleic acid from bacteria and viruses are prominent target structures that are recognized by cytoplasmic or endosomal/lysosomal PRRs with subsequent cytokine production and cellular activation, but the evidence is mounting that PRRs can also be activated by endogenous molecules.^4^ In the cytoplasm, cGAS or RIG‐I‐like receptors (RLH) recognize DNA or RNA, respectively. RIG‐I senses 5′‐triphosphorylated RNA from viral and bacterial RNA or endogenous RNA fragments generated by RNase digestion.^5^ In the endosomal/lysosomal compartment Toll‐like receptors TLR7 and TLR8 sense RNA^6^, ^7^, ^8^ in form of degradation products,^9^, ^10^ whereas TLR9 is activated by DNA with CpG sequence motif.^11^, ^12^, ^13^

Of note, most of these PRRs are highly expressed in macrophages, including tumor‐associated macrophages (TAMs), which play an integral part in shaping the tumor microenvironment. Among others, TAMs have been shown to be supportive of cancer growth, invasion, and metastasis,^14^ to be involved in establishing local T‐cell immune privilege,^15^ and to mediate resistance to radiotherapy in PDAC.^16^ Interestingly, systemic depletion of macrophages significantly diminished metastasis formation in genetically engineered mouse models of PDAC.^17^ However, PRRs are not only expressed by immune cells but also by cancer cells as well as stroma cells, thus having the potential to influence tumor biology, including therapy resistance, on multiple levels. For example, activated RIG‐I‐like helicases induce immunogenic cell death of pancreatic cancer cells and sensitize tumors toward killing by CD8^+^ T cells.^18^ In addition, TLR9 activation improves the response to radiofrequency ablation therapy in a rabbit liver cancer model.^19^ In contrast, TLR9 as well as TLR7 have initially been described to exert tumor‐promoting roles in pancreatic cancer: TLR9 ligation was demonstrated to induce pancreatic stellate cells (PSCs) to become fibrogenic and secrete chemokines that promote epithelial cell proliferation,^20^ and stimulation of TLR7 was shown to lead to an acceleration of tumor progression, while its inhibition attenuated cancer cell growth in vitro as well as in mouse models.^21^ In contrast, using syngeneic orthotopic murine tumor models, Michaelis et al. demonstrated, that treatment of tumor‐bearing mice with the TLR7/8 agonist R848 reduced tumor mass and improved survival.^22^

Here, we show that TLR7 is expressed and activated in pancreatic cancer cells in vitro and in vivo and promotes the proliferation of tumor cells. Moreover, high TLR7 expression in primary human tumor tissue samples was associated with significantly shortened survival. In contrast, global knockout of TLR7 expression in the well‐established KPC mouse model of pancreatic cancer did not result in attenuated tumor progression or increased survival of the mice. Our results thus support the notion that globally, TLR7 has no exclusive tumor‐promoting or tumor‐suppressive functions in PDAC.

## 2 METHODS

### 2.1 Patients, cell lines, and short‐term cell cultures

The human pancreatic adenocarcinoma cell lines Panc1, PaTu‐8988 T, and S2‐007 were used in this study. Panc‐1 cells and THP‐1 monocytes were obtained from the American Type Culture Collection. S2‐007 cells were from T. Iwamura^23^ (Miyazaki Medical College). PaTu‐8988 T cells were kindly provided by H. P. Elsässer (Cytobiology and Cytopathology Institute, Philipps University).

Short‐term cultures of pancreatic cancer cells from KC mice (KC623) as well as from human circulating PDAC stem cells (Lon556 and Lon560) were established as previously described.^24^

Lon556 and Lon560 were cultured in RPMI 1640 medium supplemented with 10% FCS and 0.05 mg/mL Gentamicin, the other cell lines were cultured in Dulbecco's modified eagle medium (DMEM) containing 5% FCS at 37°C and 5% (v/v) CO2.

Written informed consent was obtained from all patients prior to using tissue samples and Ethics Committee approval was available at all sites. The study was approved by the Ethics Committees at the University of Marburg and the University of Verona.

### 2.2 Construction of tissue microarrays

For the construction of tissue microarrays (TMAs), 1.0 mm sized tissue biopsies were extracted from paraffin donor blocks and transferred into pre‐punched holes on recipient paraffin blocks with a tissue microarrayer (Beecher Instruments, Inc.) equipped with a TMA booster (Alphelys). Grid layouts for tumor and normal pancreatic tissue TMAs were designed with the TMA Designer 2 software (Alphelys). The recipient blocks were sealed for 10 min at 56°C and 30 min at 4°C. This procedure was repeated twice. The TMA blocks were cut into 3.5 μm sections and placed on SuperFrost Plus slides for immunohistochemical staining.

### 2.3 Histology and immunohistochemistry

For immunohistochemistry, heat‐induced epitope retrieval was performed with citrate buffer. Staining was performed on a DAKO autostainer plus. After blocking endogenous peroxidase, sections were incubated for 45 minutes with rabbit polyclonal Anti‐TLR7 antibody (1:50; Proteintech #17232‐1‐AP) or anti‐CD68 antibody (1:100; Dako #MO876), respectively. Sections were washed and incubated with Dako REAL EnVision HRP Rabbit/Mouse polymer, which reacts with DAB‐Chromogen, according to the manufacturer's protocol. Histology and staining results were evaluated in a blinded fashion by an experienced pathologist (C. Keber). Only cases where at least 2 independent biopsy cores could be evaluated were counted. Staining intensity was graded on a scale from 0 to 3 (absent, weak, moderate, strong), and a mean score was calculated for each patient.

### 2.4 Primers, agonist, antagonist

For expression analysis following primers were used:

TLR7_h/m: FW: 5′‐CCCAGAAAATGTCCTCAACAA‐3′; RV: 5′‐ATGGTTAACCCACCAGACAAA‐3′.

RPLP0_h/m:FW: 5′‐TGGGCAAGAACACCATGATG‐3′; RV: 5’‐AGTTTCTCCAGAGCTGGGTTGT‐3′ (both primer pairs detect human and mouse transcripts simultaneously).

For TLR7 activation/inactivation the following agonist/antagonist was used: TLR7 agonist CL264 (9‐benzyl‐8 hydroxyadenine derivative containing a glycine on the benzyl group (in para); InvivoGen, cat: # tlrl‐c264e), TLR7(/9) antagonist IRS‐954 (immunoregulatory sequence; TIB Molbiol): 5′‐TGCTCCTGGAGGGGTTGT‐3′, unspecific control oligo Ctr_ODN (TIB Molbiol): 5′‐TCCTGCAGGTTAAGT‐3′.

### 2.5 Treatment with TLR7 agonist/antagonist

The different cell lines were seeded onto 6 well plates in different cell numbers depending on their cell growth (LON560: 90.000/well; KC623: 75.000/well; S2‐007: 30.000/well). KC623 cells were treated on the following day, LON560 2 days after seeding and S2‐007 was pre‐incubated 1 day in a serum‐free medium before treatment was added. On the day of the treatment, the cells were washed once with PBS, the medium was changed to medium with reduced serum concentration (0%‐ or 1%‐serum as indicated in the respective figure), and CL264 or IRS‐954/Ctr_ODN was added.

### 2.6 Cell viability assays

Cell viability was measured by MTT assay as described previously.^25^, ^26^ Briefly, after 72 h of incubation, cells were incubated for 1–2 h with MTT‐reagent (thiazolyl blue, Carl Roth GmbH) at 37°C, solubilized and measured at 570 nm with the Multiskan FC photometer (Thermo Scientific).

### 2.7 Protein extraction and Western Blot analyses

The following antibodies were used for western blot analyses: anti–Cyclin D1 (Abcam, cat. # ab16663); anti‐p21 (Cell Signaling, cat. # 2947); anti‐PARP (Cell Signaling, cat. # 9532); anti–caspase‐3 (Cell Signaling, cat. # 9664 and 9665); anti–actin, HRP coupled (Sigma‐Aldrich, cat. # A3854); anti‐TLR7 (Proteintech cat. #17232‐1‐AP).

For protein extraction, cells were collected together with medium and centrifuged at 1600 rpm at 4°C for 5 minutes. Pellets were washed twice with ice‐cold PBS and then resuspended in 200 ml lysis buffer (PBS containing protease inhibitors (Protease Arrest, GBiosciences) and phosphatase inhibitors (PMSF 1 mmol/L, EDTA 0.5 mmol/L, sodium pyrophosphate 25 mmol/L, sodium orthovanadate, 10 mmol/L, sodium fluoride 50 mmol/L)). Cells were sonicated (LabSonic, BBraun) and protein content was assessed using Protein Assay Reagent (Thermo Scientific). For Western blotting, 15 mg proteins were electrophoresed on SDS‐polyacrylamide gels and electrophoretically transferred onto nitrocellulose membranes (Optitran, GE Healthcare Life Sciences). Membranes were blocked in 5% nonfat dry milk in TBST (10 mmol/L Tris–HCl, pH 7.6, 100 mmol/L NaCl, 0.1% Tween 20) for 2 hours at room temperature and then probed with appropriate antibodies.

### 2.8 Animal experiments

Mice were maintained in IVCs in a climate‐controlled room kept at 22°C, exposed to a 12:12‐hour light–dark cycle, fed standard laboratory chow, and given water ad libitum.

The *TLR7* ^*−/−*^ mouse strain as well as the *LsL‐Kras* ^*G12D*^; *Pdx1‐Cre* double mutant (“KC mice”) and *LsL‐Kras* ^*G12D*^; *LsL‐Trp53* ^*R172H*^; *Pdx1‐Cre* triple mutant (“KPC mice”) strains have been described previously.^27^, ^28^ All mouse strains were originally on a mixed 129/SvJ/C57Bl/6 background but were backcrossed to a pure C57Bl/6 background for at least six generations. Mutant strains were intercrossed to produce cohorts with the genotypes indicated in the manuscript.

Upon signs of terminal illness, such as weight loss, diminished activity, and/or abdominal bloating due to ascites, mice were euthanized and the pancreas was removed, inspected for grossly visible tumors, and preserved in 4% formalin solution (Otto Fischar GmbH). All animals that were found to have invasive adenocarcinomas of the pancreas upon necropsy were included as events. Animals that died of other causes, as determined by histologic evaluation of pancreata after necropsy, as well as animals that were still alive at the time of evaluation, were censored.

All animal procedures were ethically reviewed and approved by Regierungspräsidium Gießen (Germany) and all experiments were performed in accordance with the European guidelines for the care and use of laboratory animals confirming Directive 2010/63/EU.

### 2.9 GO enrichment analysis of tumor material from KPC mice

Whole transcriptome sequencing was performed on FFPE (formalin‐fixed paraffin‐embedded) tissue from WT KPC (*n* = 9) and TLR7^−/−^ KPC (*n* = 10) pancreata by a commercial service (CeGaT GmbH, Tübingen, Germany) and reads mapped to a standard genome.^29^ Normalized read counts were used to identify differentially expressed genes using a 10% false discovery rate as a threshold, and the resulting list was analyzed for significantly enriched gene sets using the PANTHER Classification System (pantherdb.org ^30^).

### 2.10 Software

To compute and compare survival rates between mouse cohorts as well as to analyze patient clinicopathological data and generate Kaplan–Meier curves, the GraphPad Prism 9 program (GraphPad Software) was used.

## 3 RESULTS

### 3.1 TLR7 is expressed in human pancreatic cancer cells in vivo and in vitro

**FIGURE 1:**
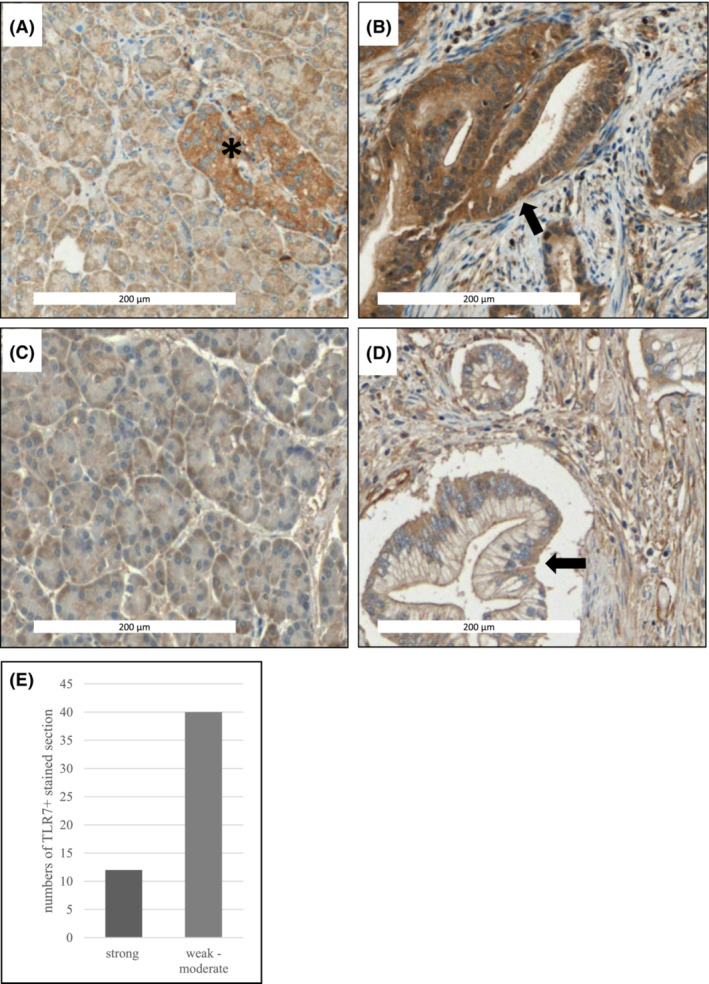
Expression of TLR7 in primary human tissues. (A–D) Tissue microarrays (TMAs) of human PDAC (B, D) and corresponding normal (A, C) tissue, were stained against TLR7 (Proteintech #17232‐1‐AP), results showing strong (B) and moderate (D) expression of TLR7 in tumor cells (arrows). Islets of Langerhans (A, asterisk) also stained positive for TLR7. E: Quantification of staining results from TMA sections.

**FIGURE 2:**
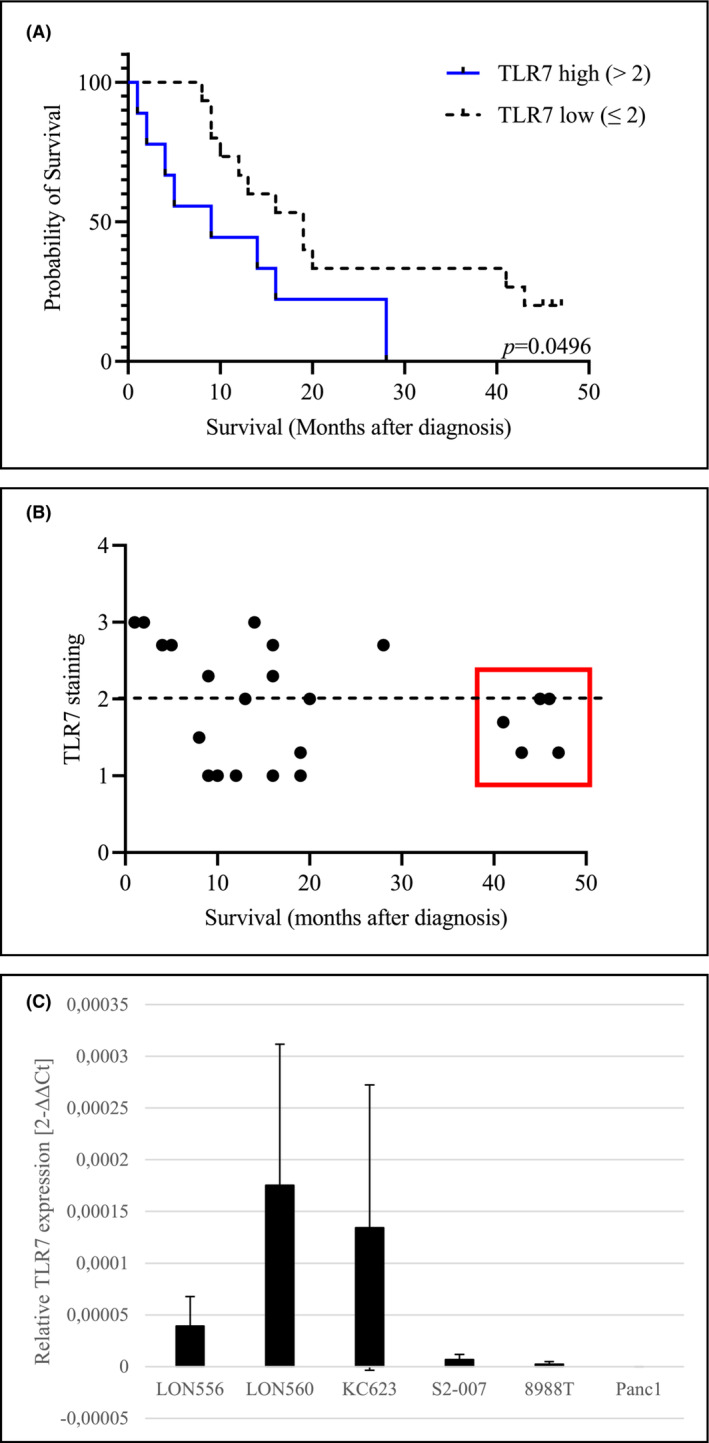
Correlation of TLR7 expression with patient survival and relative age of cell cultures. (A) Kaplan–Meier curves of overall survival according to TLR7 IHC (censored data are flagged). *n* = 24; Log‐rank (Mantel‐Cox) test. (B) Correlation of survival and TLR7 expression reveals that all long‐time survivors (<36 months from diagnosis) exhibit weak to moderate TLR7 staining. (C) Q‐RT‐PCR analyses of TLR7 mRNA expression in short‐term cultures upon thawing in comparison to established PDAC cell lines. Expression values are shown relative to the housekeeping gene RPLP0 in each cell line. Bars represent the mean ± SDM of two to five independent experiments.

Staining of tissue microarray (TMA) sections of human primary cases of PDAC (*n* = 52) with TLR7‐specific antibodies revealed that TLR7 is strongly overexpressed in 23% (*n* = 12) and weakly/moderately expressed in 77% (*n* = 40) of cases (Figure 1B,D & E). Staining of normal exocrine tissue was uniformly weak (Figure 1A & C), while normal pancreatic islets showed moderate staining intensities (Figure 1A). A comparison of clinicopathological features with TLR7 expression data revealed no statistically significant correlation of TLR7 expression status with tumor grade, stage, or TNM status (Table 1). However, high TLR7 expression in the tumor correlated with statistically significantly reduced overall survival (Figure 2A) with all long‐time survivors (>36 months after diagnosis) showing weak to moderate TLR7 expression (Figure 2B).

**TABLE 1:** Summary of clinicopathological data of primary human PDAC tissue donors. From originally 76 cases, 62 cases with at least 2 out of 3 TMA sections available were analyzed. Of these 10 cases were classified as intraductal papillary mucinous neoplasm (IPMN) and therefore excluded from analyses. The remaining cases were categorized into two groups, depending on the mean TLR7 staining intensity of the respective sections (weak to moderate or strong). The distribution of parameters was analyzed by Chi^2^ Test of Trend showing no significant differences between groups

Variable	Cases	TLR7 expression				*p*‐value
		weak/moderate		strong		Chi^2^ Test of Trend
		*n*		*n*		
TLR7 staining	52	40	77%	12	23%	
Clinical Stage (AJCC)	45	35		10		
IA	1	1	100%	0	0%	0.83
IB	14	10	71%	4	29%	
IIA	7	6	86%	1	14%	
IIB	23	18	78%	5	22%	
Tumor Size	47	36		11		0.59
<2 cm	4	4	100%	0	0%	
2–4 cm	30	22	73%	8	27%	
>4 cm	13	10	77%	3	23%	
Tumor Grade	45	35		10		0.87
G1	1	1	100%	0	0%	
G2	36	27	75%	9	25%	
G3	7	7	100%	0	0%	
G4	1	0	0%	1	100%	
% of cases showing	42	33		9		
Lymphatic invasion	6	5	83%	1	17%	
Vascular invasion	5	4	80%	1	20%	
Perineural invasion	28	21	75%	7	25%	

Quantitative real‐time PCR analyses showed that TLR7 was highly expressed at the mRNA level in short‐term cultures from both, human (LON560) as well as murine (KC623) PDAC tumor cells. In contrast, expression was low or absent in established long‐term cultured cell lines derived both from liver metastases (PaTu‐8988 T, S2‐007) as well as from primary tumors (Panc‐1) (Figure 2C). For verification real‐time PCR analysis was repeated in the presence of TLR7‐positive THP‐1 monocytic cells (Figure S1A).

### 3.2 Inhibition of TLR7 reduces cell viability in vitro

**FIGURE 3:**
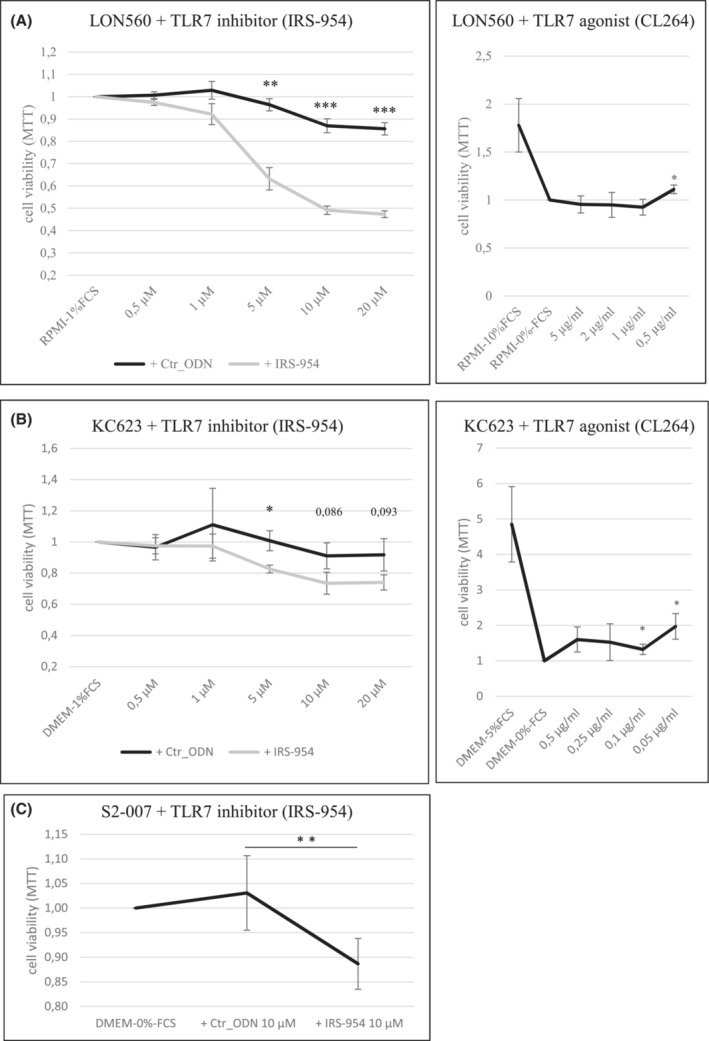
TLR7 mediates growth‐stimulatory effects in cancer cells in vitro. (A & B) LON560 (A) and KC623 (B) were treated with different concentrations of IRS‐954/Ctr_ODN [0.5 μM–20 μM] or CL264 [5 μg/mL–0.5 μg/mL or 0.5 μg/mL–0.05 μg/mL]. Cell viability was measured by MTT assay 72 h after treatment. (C) S2‐007, a low‐expressing TLR7 cell line, was treated with 10 μM IRS‐954/Ctr_ODN (concentration with the highest effect in LON560). Cell viability was measured by MTT assay 72 h after treatment. Bars represent the mean ± SDM of at least three independent experiments normalized to control cells (0%‐FCS/ 1%‐FCS). Ctr_ODN, control oligo. (Students *t* test: *p < 0.05, **p < 0.01, ***p < 0.001).

To investigate the functional role of TLR7 expression in pancreatic cancer cells, we treated TLR7‐high human Lon560 and murine KC623 cells with the well‐established inhibitor IRS‐954. Inhibition of TLR7 led to strongly reduced cell viability in LON560 cells (Figure 3A, left panel). This effect was less pronounced, but also clearly apparent in KC623 cells (Figure 3B, left panel). Conversely, treatment of the TLR7‐low S2‐007 cells with the same inhibitor at a high dose showed only a mild impact on cell viability (Figure 3C). Functionally, inhibition of TLR7 through IRS‐954 did not lead to reduced mRNA levels neither in cancer cells nor in THP‐1 monocytic control cells (Figure S1B).

Interestingly, treatment with the TLR7 agonist CL264 had little or no effect on LON560 cells even in the absence of any other potential stimulants (serum‐free culture conditions) (Figure 3A, right panel), suggesting that TLR7 activity was already maximally stimulated by endogenous ligands in these cells. KC623 cells showed moderate growth‐stimulatory effects after CL264 treatment (Figure 3B, right panel), correlating with the less pronounced growth inhibition by the IRS‐954 inhibitor.

In order to test whether reduced viability was due to the induction of apoptosis or activation of classical cell cycle checkpoints, we performed Western blot analyses of typical markers for both processes. However, neither PARP nor Caspase‐3 cleavage as markers of apoptosis induction, nor regulation of cell growth‐associated proteins Cyclin D1 and p21 as typical markers of cell cycle arrest were apparent following TLR7‐inhibition, suggesting that reduced viability is the consequence of more complex regulatory mechanisms(Figure S2).

### 3.3 Global TLR7 deficiency does not attenuate tumor progression in transgenic mouse models of pancreatic cancer

**FIGURE 4:**
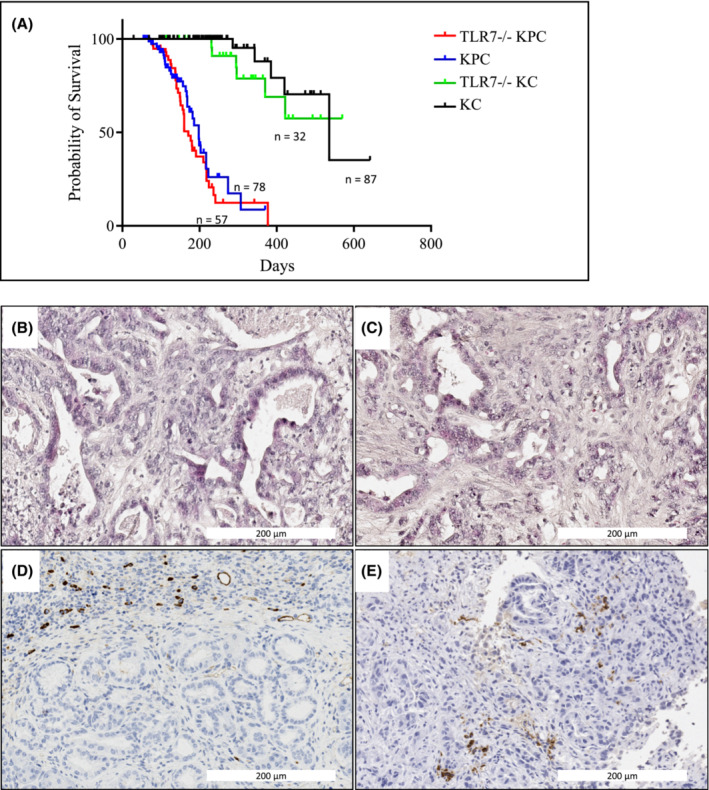
Global TLR7 knockout does not attenuate tumor progression in vivo. (A) Kaplan–Meier analyses revealed that TLR7 deficiency did not result in any survival benefit, neither in the KPC nor in the KC mouse model. (B & C) Representative histological sections (H&E staining) of 7‐month‐old WT‐KPC (B) and TLR7^−/−^‐KPC (C) mice. (D & E) Representative images of CD68 staining for monocytes in 7‐month‐old WT‐KPC (D) and TLR7^−/−^‐KPC (E) mice.

In order to evaluate the dependency of pancreatic tumorigenesis on TLR7 expression in vivo, we crossed mice with a global TLR7 knockout (TLR7^−/−^ mice) with mice of the KC and KPC strains. As shown in Figure 4A, TLR7 status did not have any discernable influence on the survival of KPC mice, with a median survival of 171 days in the TLR7^−/−^ cohort compared to 198 days in KPC WT mice. In the less severe KC tumor mouse model, TLR7^−/−^ KC mice even tended to display a trend to shorter survival (Figure 4A), although it should be noted that many animals from this cohort had to be sacrificed for reasons unrelated to pancreatic tumorigenesis (mainly development of papillomas and lymphomas), which may be attributable to impaired restriction of endogenous retrovirus (ERV) activation as previously reported for TLR7^−/−^ mice.^31^

Blinded evaluation of histological features of end‐stage tumors from KPC mice demonstrated considerable inter‐tumoral heterogeneity in features such as tumor cell differentiation, stroma content, or lymphocyte infiltration, but did not reveal systematic differences between tumors from TLR7^−/−^ or TLR7^wt^ KPC mice (Figure 4B, C). Likewise, there was no systematic difference in the presence of monocytic cells in TLR7^−/−^ or TLR7^wt^ KPC tumors as evaluated by CD68 staining of representative tumor sections (Figure 4D, E).

**FIGURE 5:**
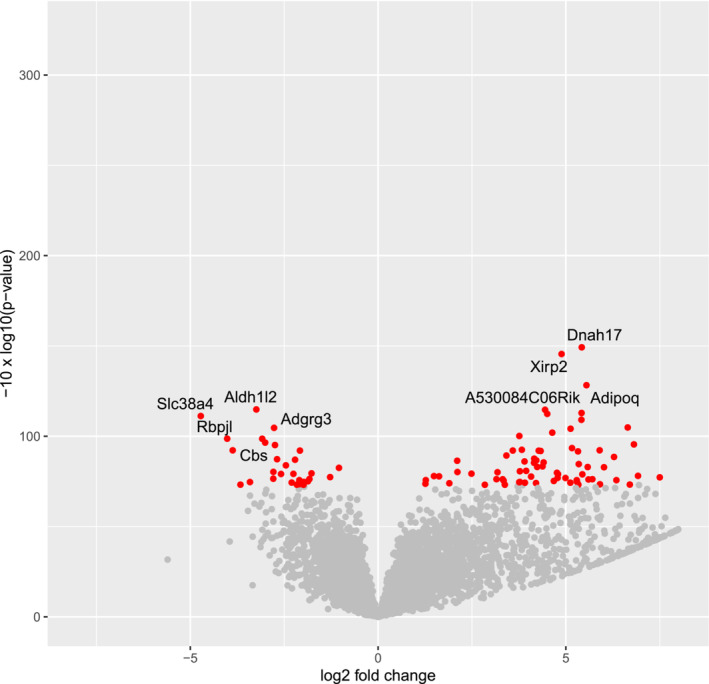
Volcano plot of differentially expressed genes. The log2 FC (fold change) indicates the mean expression level for each gene. Positive values denote genes upregulated in TLR7 KO vs. control KPC tumors; negative values denote genes downregulated in TLR7 KO tumors. Each dot represents one gene; red dots denote genes significantly differentially expressed at a false discovery rate (FDR) of 10%.

In an effort to gain further insights into potential systematic changes on the RNA level, we performed RNAseq analysis of bulk tumor material from TLR7^−/−^ (*n* = 10) and TLR7^wt^ (*n* = 9) KPC mice (Figure 5). Statistical analyses revealed a relatively small number of significantly differentially expressed genes (*n* = 99 at 10% false discovery rate; Table 2), which may again be reflective of relatively large intertumoral heterogeneity within both cohorts. Interestingly, however, gene set enrichment analysis of the sequencing data using the PANTHER Classification System revealed that a large number of GO terms related to immune system functions, such as “leukocyte activation (GO:0045321),” “lymphocyte activation (GO:0046649),” “positive regulation of innate immune response (GO:0045089)” etc, which were significantly enriched among the set of regulated genes (Table 3), hinting at systematic changes in the immune landscape of the tumors in response to the TLR7 deficiency.

**TABLE 2:** List of differentially expressed genes. TLR7 knockout (*n* = 10) and control KPC tumors (*n* = 9) were subjected to RNAseq analysis and readcounts were determined using HTseq‐count. Listed are genes that were statistically significantly differentially expressed at a false discovery rate (FDR) of 10%. Positive values denote genes upregulated in TLR7 KO versus control KPC tumors; negative values denote genes downregulated in TLR7 KO tumors

Gene name	Description	log2 Fold Change	padj
Vmn1r181	Vomeronasal 1 receptor 181	23.432	1.17 E‐10
Xirp2	Xin Actin‐binding repeat containing 2	4.886	0.0024
Dnah17	Dynein, axonemal, heavy chain 17	5.423	0.0024
Adipoq	Adiponectin, C1Q, and collagen domain containing	5.549	0.0099
Aldh1l2	Aldehyde dehydrogenase 1 family, member L2	−3.242	0.0243
A530084C06R	RIKEN cDNA A530084C06 gene	4.446	0.0243
Snx22	Sorting nexin 22	4.504	0.0243
Ccdc63	Coiled‐coil domain containing 63	5.418	0.0243
Slc38a4	Solute carrier family 38, member 4	−4.721	0.0244
Samd15	Sterile alpha motif domain containing 15	5.414	0.0270
Adgrg3	Adhesion G protein‐coupled receptor G3	−2.770	0.0340
Ccdc106	Coiled‐coil domain containing 106	5.126	0.0340
Majin	Membrane‐anchored junction protein	6.647	0.0340
Rimklb	Ribosomal modification protein rimk‐like family member B	4.638	0.0393
Fanci	Fanconi anemia, complementation group I	3.761	0.0441
Rbpjl	Recombination signal binding protein for immunoglobulin kappa J	−4.018	0.0454
Cbs	Cystathionine beta‐synthase	−3.085	0.0454
Cfap54	Cilia and flagella associated protein 54	−3.007	0.0530
Slc38a3	Solute carrier family 38, member 3	−3.871	0.0536
Nr5a2	Nuclear receptor subfamily 5, group A, member 2	−2.745	0.0536
Adgb	Androglobin	−2.081	0.0536
C130026I21Ri	RIKEN cDNA C130026I21 gene	3.587	0.0536
Gm10718	Predicted gene 10,718	3.828	0.0536
Trpm3	Transient receptor potential cation channel, subfamily M, member 3	4.277	0.0536
Plin1	Perilipin 1	4.329	0.0536
Iqsec3	IQ motif and Sec7 domain 3	5.163	0.0536
Tmigd1	Transmembrane and immunoglobulin domain containing 1	5.323	0.0536
Cacna2d3	Calcium channel, voltage‐dependent, alpha2/delta subunit 3	5.899	0.0536
Gm11127	Predicted gene 11,127	6.812	0.0536
Btla	B and T lymphocyte associated	3.417	0.0652
Ndp	Norrie disease (pseudoglioma)	6.283	0.0682
Cpn1	Carboxypeptidase n, polypeptide 1	−2.690	0.0725
Zfp236	Zinc finger protein 236	−2.211	0.0725
Gata3	GATA binding protein 3	2.106	0.0725
Abcc2	ATP‐binding cassette, sub‐family C (CFTR/MRP), member 2	4.157	0.0725
Gm10801	Predicted gene 10,801	4.213	0.0725
Olfr639	Olfactory receptor 639	3.900	0.0733
Foxp3	Forkhead box P3	4.152	0.0748
Olfr961	Olfactory receptor 961	4.408	0.0748
Aire	Autoimmune regulator	5.344	0.0784
Srrm4	Serine/arginine repetitive matrix 4	−2.454	0.0821
Spock3	Sparc/osteonectin, cwcv, and kazal‐like domains proteoglycan 3	4.236	0.0831
Scrt1	Scratch family zinc finger 1	4.380	0.0831
Ppp1r1c	Protein phosphatase 1, regulatory inhibitor subunit 1C	5.581	0.0831
Vmn1r197	Vomeronasal 1 receptor 197	6.015	0.0831
Appl2	Adaptor protein, phosphotyrosine interaction 2	−1.041	0.0840
Glt1d1	Glycosyltransferase 1 domain containing 1	−2.788	0.0953
Ptprn2	Protein tyrosine phosphatase, receptor type, N polypeptide 2	−2.584	0.0953
Wnk2	WNK lysine deficient protein kinase 2	−2.254	0.0953
Gpt	glutamic pyruvic transaminase, soluble	−1.772	0.0953
H2ax	H2A.X variant histone	2.113	0.0953
Chst1	Carbohydrate sulfotransferase 1	2.487	0.0953
Tlr1	Toll‐like receptor 1	3.180	0.0953
Il12rb2	Interleukin 12 receptor, beta 2	3.775	0.0953
Rgs20	Regulator of G‐protein signaling 20	3.943	0.0953
Gm11032	Predicted gene 11,032	4.763	0.0953
Trim66	Tripartite motif‐containing 66	4.791	0.0953
M1ap	Meiosis 1‐associated protein	5.439	0.0953
Pah	Phenylalanine hydroxylase	−3.665	0.0996
Lrrc7	Leucine‐rich repeat containing 7	−3.411	0.0996
Adm2	Adrenomedullin 2	−2.791	0.0996
Prox1	prospero homeobox 1	−2.300	0.0996
Reps2	RALBP1‐associated Eps domain containing protein 2	−2.163	0.0996
Sox6	SRY (sex‐determining region Y)‐box 6	−2.097	0.0996
Gls2	Glutaminase 2 (liver, mitochondrial)	−2.086	0.0996
Ipo11	Importin 11	−1.985	0.0996
Unc5a	Unc‐5 netrin receptor A	−1.976	0.0996
Cntfr	Ciliary neurotrophic factor receptor	−1.868	0.0996
Adk	Adenosine kinase	−1.828	0.0996
Rora	RAR‐related orphan receptor alpha	−1.277	0.0996
Kcnd1	Potassium voltage‐gated channel, shal‐related family, member 1	1.261	0.0996
Ppp1r18	Protein phosphatase 1, regulatory subunit 18	1.271	0.0996
Sfxn3	Sideroflexin 3	1.488	0.0996
Pik3ip1	phosphoinositide‐3‐kinase interacting protein 1	1.623	0.0996
Irx3	Iroquois‐related homeobox 3	1.897	0.0996
Rell2	RELT‐like 2	2.843	0.0996
Catip	Ciliogenesis‐associated TTC17 interacting protein	3.152	0.0996
Nanos1	Nanos C2HC‐type zinc finger 1	3.318	0.0996
Rasd2	RASD family, member 2	3.343	0.0996
Cfd	Complement factor d (adipsin)	3.376	0.0996
Gm10800	Predicted gene 10,800	3.759	0.0996
Cpne9	Copine family member ix	3.777	0.0996
Phex	Phosphate regulating endopeptidase homolog, x‐linked	3.910	0.0996
Kcnb2	Potassium voltage‐gated channel, shab‐related subfamily, member 2	4.074	0.0996
Prss57	Protease, serine 57	4.205	0.0996
Pou3f1	POU domain, class 3, transcription factor 1	4.680	0.0996
Gm14226	Predicted gene 14,226	4.780	0.0996
Kcnip4	Kv channel interacting protein 4	4.990	0.0996
Trhde	TRH‐degrading enzyme	5.120	0.0996
Tex15	Testis expressed gene 15	5.290	0.0996
Ces1f	Carboxylesterase 1F	5.299	0.0996
Apoa1	Apolipoprotein A‐I	5.333	0.0996
Vmn1r60	Vomeronasal 1 receptor 60	5.602	0.0996
Gm10720	Predicted gene 10,720	5.707	0.0996
Slc6a19	Solute carrier family 6 (neurotransmitter transporter), member 19	5.908	0.0996
Myom2	Myomesin 2	6.346	0.0996
Olfr1238	Olfactory receptor 1238	6.703	0.0996
Usp17la	Ubiquitin‐specific peptidase 17‐like A	6.921	0.0996
Gpr37l1	G protein‐coupled receptor 37‐like 1	7.500	0.0996

**TABLE 3:** List of GO terms significantly enriched among differentially expressed genes. Differentially expressed genes (Table 2) were analyzed for significantly enriched gene sets using the PANTHER Classification System. Interestingly upregulated genes in TLR7 knockout tumors (+) clustered around immune system‐related GO terms

GO biological process complete	Regul.	*p*‐value	fdr
Chromatin organization involved in the regulation of transcription (GO:0034401)	+	3.17 E‐08	4.90 E‐04
DNA packaging (GO:0006323)	+	4.94 E‐07	3.81 E‐03
Leukocyte activation (GO:0045321)	+	6.52 E‐07	3.36 E‐03
Regulation of lymphocyte activation (GO:0051249)	+	8.86 E‐07	3.42 E‐03
Cell activation (GO:0001775)	+	1.42 E‐06	4.38 E‐03
Regulation of leukocyte activation (GO:0002694)	+	2.29 E‐06	5.89 E‐03
Regulation of cell activation (GO:0050865)	+	3.32 E‐06	7.32 E‐03
Immune system process (GO:0002376)	+	4.00 E‐06	7.72 E‐03
Positive regulation of gene expression. epigenetic (GO:0045815)	+	4.49 E‐06	7.70 E‐03
Lymphocyte activation (GO:0046649)	+	5.23 E‐06	8.07 E‐03
Regulation of immune system process (GO:0002682)	+	5.38 E‐06	7.54 E‐03
Translation (GO:0006412)	+	7.39 E‐06	9.51 E‐03
Chromatin organization involved in negative regulation of transcription (GO:0097549)	+	7.58 E‐06	8.99 E‐03
Cellular amino acid metabolic process (GO:0006520)	−	8.07 E‐06	8.89 E‐03
Chromatin assembly (GO:0031497)	+	9.55 E‐06	9.82 E‐03
Long‐chain fatty acid metabolic process (GO:0001676)	+	1.50 E‐05	1.44 E‐02
Peptide biosynthetic process (GO:0043043)	+	2.53 E‐05	2.30 E‐02
Positive regulation of innate immune response (GO:0045089)	+	2.64 E‐05	2.26 E‐02
Olefinic compound metabolic process (GO:0120254)	+	2.79 E‐05	2.26 E‐02
Regulation of interferon‐gamma production (GO:0032649)	+	2.80 E‐05	2.16 E‐02
Regulation of gene expression. epigenetic (GO:0040029)	+	3.40 E‐05	2.50 E‐02
Chromatin assembly or disassembly (GO:0006333)	+	4.13 E‐05	2.90 E‐02
Positive regulation of immune system process (GO:0002684)	+	5.98 E‐05	4.01 E‐02
Selective autophagy (GO:0061912)	−	6.09 E‐05	3.92 E‐02
T Cell activation (GO:0042110)	+	6.25 E‐05	3.86 E‐02
Alpha‐amino acid metabolic process (GO:1901605)	−	7.00 E‐05	4.15 E‐02
Regulation of lymphocyte proliferation (GO:0050670)	+	8.00 E‐05	4.57 E‐02
Regulation of mononuclear cell proliferation (GO:0032944)	+	8.06 E‐05	4.44 E‐02

Abbreviations: fdr, false discovery rate; regul., regulation.

## 4 DISCUSSION

In the course of developing immunotherapeutic approaches for the treatment of malignant tumors, TLR agonists are already used in clinical settings to induce anti‐tumor immunity.^32^, ^33^, ^34^, ^35^ In this context, TLRs have also been proposed as potential targets in the therapy of PDAC, but studies addressing the biology and clinical utility of TLRs in general and TLR7, in particular, remain scarce.

Previous studies resulted in conflicting reports regarding the expression pattern of TLR7 in normal and neoplastic human pancreas tissue. While Ochi et al. reported that expression was completely absent from healthy pancreas but strongly present in both epithelial as well as stromal cells in PDAC,^21^ Helminen et al. described the distinct expression of TLR7 (as well as TLR8) in beta cells within pancreatic islets.^36^ Our own results confirm the data of Helminen et al., clearly showing robust expression of TLR7 in islets of Langerhans. This result can be taken as further evidence of the fact that the role of pattern recognition receptors extends beyond their functions in the innate immune system, both in physiological as well as pathophysiological contexts.

Our data further confirm that TLR7 is expressed by tumor cells within primary human tumor tissue^21^, ^22^, ^37^ and high TLR7 expression seems to correlate with shorter overall survival which, to our knowledge, has not been reported before. Although we could not observe any correlation between tumor stage and TLR7 expression as Grimmig et al. reported, analyses of our clinicopathological data uncovered a group of long‐time surviving patients showing exclusively weak to moderate TLR7 expression.

In the course of this study, we further identified a subset of TLR7 expressing pancreatic cancer cell lines. Interestingly, short‐term cultured cells from both human as well as mouse tumors showed considerably higher TLR7 expression than high‐passage established cell lines, indicating that TLR7 expression is not selected for under standard culture conditions and may be lost over time. Stimulation of TLR7‐positive cells with a TLR7‐specific agonist did not significantly alter cell growth, which is consistent with previous results showing that TLR7 ligation with ssRNA40 did not have any direct proliferative effects on transformed epithelial cells from KC mice and did not affect their viability.^21^ Conversely, inhibition of TLR7 in the same cell lines reduced cell viability in a dose‐dependent manner, indicating that TLR7 is not only expressed and functional in the tumor cells but also active, which hints at the presence of effective endogenous ligands and autocrine signaling mechanisms.

Two studies have previously investigated the role of TLR7 in promoting or inhibiting pancreatic tumor progression in vivo, reporting partly conflicting results. The first one^21^ reported that treatment of p48Cre; Kras^G12D^ (KC) mice with the TLR7 agonist ssRNA40 strongly accelerated tumor progression, while TLR7 inhibition with IRS‐954 was able to block caerulein‐induced inflammation‐mediated tumor progression in KC mice. Effects of TLR7 inhibition on the natural course of disease (unstimulated by caerulein‐induced inflammation) in KC or KPC mice were not reported.

In contrast, the second study^22^ reported that the TLR7 agonist R848 elicited strong anti‐tumor responses in syngeneic orthotopic murine PDAC models and protected the animals from cachexia manifestations. The authors concluded that anti‐tumor effects of R848 were mediated by host‐derived TLR7‐positive stromal cells, rather than by direct influence on neoplastic cells since the implantation of syngeneic tumor cells in TLR7^−/−^ host mice with subsequent R848 treatment did not result in attenuated, but instead in accelerated tumor growth.

Of note, although both studies made use of the KC, KPC, and TLR7^−/−^ mouse strains also used in our study, neither study reported crossing the KC and/or KPC mouse model(s) onto the TLR7 knockout background. Instead, results were obtained by different combinations of a tumor cell or bone marrow cell grafting and treatment with TLR7 agonists or inhibitors. It is thus difficult to determine how faithfully either of these studies reflected the in vivo situation of pancreatic tumorigenesis, and in how far off‐target effects or the pharmacodynamic properties of agonists and inhibitors influenced the study results. Our own results surprisingly demonstrated neither a protective nor a tumor‐promoting net effect of TLR7 ablation in vivo, although tumor‐promoting functions in vitro were readily apparent. This may be reflective of opposing roles of TLR7 functions in tumor cells versus cells of the inflammatory stroma, as can also be concluded from the observation of Michaelis et al. that tumor‐attenuating effects of TLR7 agonist treatment were lost in TLR7^−/−^ mice and instead resulted in increased growth of TLR7‐competent tumor cells in TLR7‐deficient host animals.^22^ Our RNAseq analyses indicate that despite considerable inter‐tumoral heterogeneity in the KPC mouse model, TLR7 deficiency systematically alters the immune landscape in PDAC tumors. For instance, several genes known to play central roles in T cell biology, including transcription factors GATA3, Foxp3, and AIRE as well as the Interleukin12 receptor beta2, but also the B‐ And T‐Lymphocyte Attenuator BTLA were significantly upregulated in TLR7^−/−^ tumors. On the contrary, the B cell regulator ADRGRG3 as well as the transcription factor RBPJ, has been shown to be a critical factor in T‐helper (TH) subset polarization (https://doi.org/10.1038/s41467‐019‐09276‐w), were found to be strongly downregulated in TLR7^−/−^ tumors. Of note is also the upregulation of the TLR1 receptor in response to the absence of TLR7 expression, although TLR1 and TLR7 sense very different sets of biomolecules. The profound impact of TLR7 deficiency on the immune landscape of pancreatic tumors is also reflected by the results of our gene set enrichment analyses which revealed that a large number of GO terms related to immune system functions, such as “leukocyte activation (GO:0045321),” “lymphocyte activation (GO:0046649),” “positive regulation of innate immune response (GO:0045089),” were significantly overrepresented among the differentially regulated genes. However, given the complex interplay of different types of immune and stroma cells in the tumor microenvironment and the wide range of subtypes and functional states of different immune cell populations, much more in‐depth work will be required to precisely dissect the roles of TLR7 and its ligands in different populations of epithelial and stromal cells and to understand their relative contributions to the promotion or attenuation of tumor progression in pancreatic cancer.

## AUTHOR CONTRIBUTIONS

**Maren Stark:** Data curation (equal); formal analysis (equal); investigation (equal); visualization (equal); writing – original draft (equal). **Marina Nicolai:** Data curation (equal); formal analysis (equal); investigation (equal); visualization (equal); writing – original draft (equal); writing – review and editing (equal). **Marina Tatura:** Data curation (equal); formal analysis (equal); investigation (equal); visualization (equal); writing – original draft (equal); writing – review and editing (equal). **Corinna U. Keber:** Data curation (equal); formal analysis (equal); investigation (equal); visualization (equal); writing – review and editing (equal). **Andreas Kaufmann:** Data curation (equal); formal analysis (equal); investigation (equal); visualization (equal); writing – review and editing (equal). **Ho‐Ryon Chung:** Formal analysis (equal); visualization (equal); writing – review and editing (equal). **Emily P. Slater:** Data curation (equal); investigation (equal); writing – review and editing (equal). **Christopher Heeschen:** Formal analysis (equal); visualization (equal); writing – review and editing (equal). **Rita T Lawlor:** Data curation (equal); formal analysis (equal); investigation (equal); visualization (equal); writing – review and editing (equal). **Aldo Scarpa:** Formal analysis (equal); visualization (equal); writing – review and editing (equal). **Detlef K. Bartsch:** Data curation (equal); funding acquisition (equal); investigation (equal); resources (equal); writing – review and editing (equal). **Thomas Gress:** Conceptualization (equal); funding acquisition (equal); resources (equal); supervision (equal); visualization (equal); writing – review and editing (equal). **Stefan Bauer:** Conceptualization (equal); formal analysis (equal); funding acquisition (equal); project administration (equal); resources (equal); supervision (equal); visualization (equal); writing – review and editing (equal). **Malte Buchholz:** Conceptualization (equal); formal analysis (equal); funding acquisition (equal); project administration (equal); resources (equal); supervision (equal); visualization (equal); writing – review and editing (equal).

## FUNDING INFORMATION

This work was funded in part by funds obtained from the German Research Foundation (DFG) (clinical research unit “Clinical relevance of tumor‐microenvironment interactions in pancreatic cancer” ‐ DFG‐KFO325) to DKB, TMG, SB, and MB.

## CONFLICT OF INTEREST

The authors declare no competing financial interests.

## DATA AVAILABILITY

Data are available on request from the authors.

## Supporting information


Figure S1.

Figure S2.
