# Supplementary material for: Dissecting the role of toll‐like receptor 7 in pancreatic cancer
Source: Cancer Med. 2023 Jan 5;12(7):8542–56. doi: 10.1002/cam4.5606 (PMC10134280; doi:10.1002/cam4.5606)
Supplement: Supplementary file 1 — Figure S1. [file CAM4-12-8542-s002.pdf]

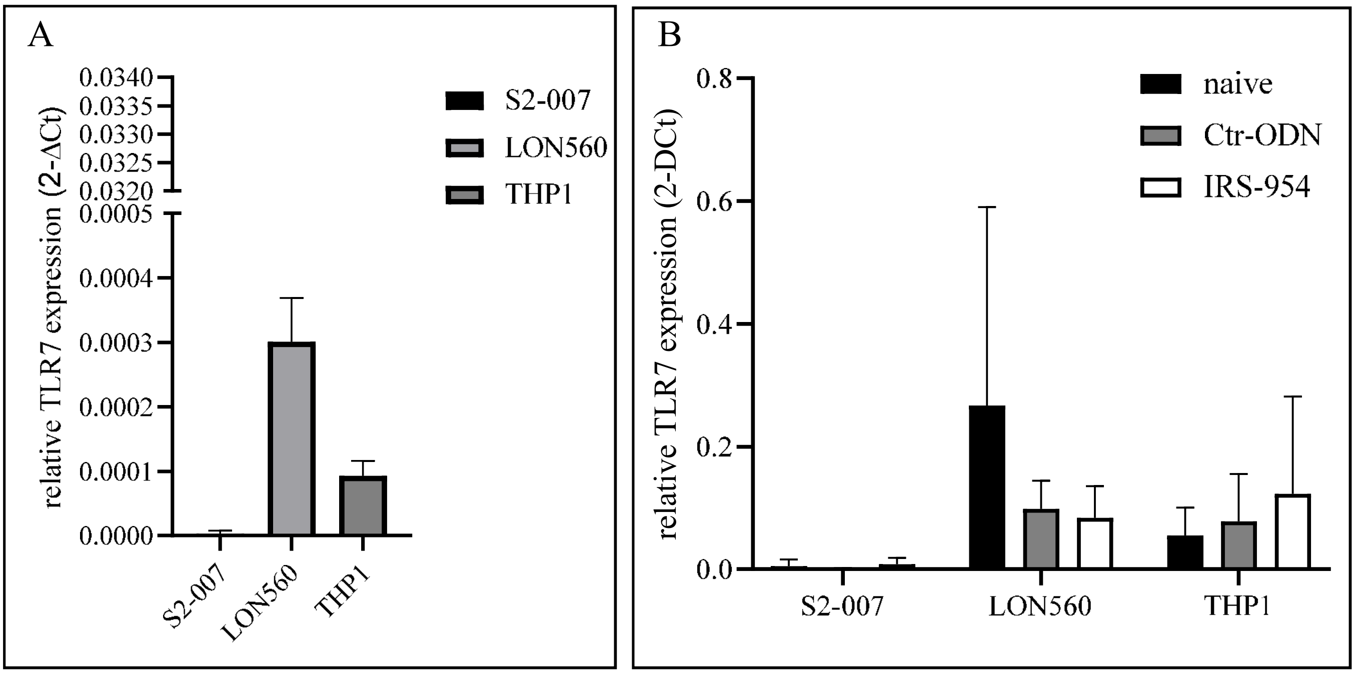

Supplementary Fig. 1: TLR7 expression in pancreatic cancer and TLR7 positive control cell lines A: TLR7 expression of naïve S2-007, LON560 and THP-1 cells (n=5) was measured by quantitative realtime PCR. B: S2-007, LON560 and THP1 moncytic cells were treated with 10 µM IRS-954 or Ctr_ODN (n = 5). TLR7 mRNA expression was measured 72h after treatment by quantitative realtime PCR. Bars represent mean TLR7 expression ±SDM relative to housekeeping gene RPLP0.
