# Supplementary material for: Dissecting the role of toll‐like receptor 7 in pancreatic cancer
Source: Cancer Med. 2023 Jan 5;12(7):8542–56. doi: 10.1002/cam4.5606 (PMC10134280; doi:10.1002/cam4.5606)
Supplement: Supplementary file 2 — Figure S2. [file CAM4-12-8542-s001.pdf]

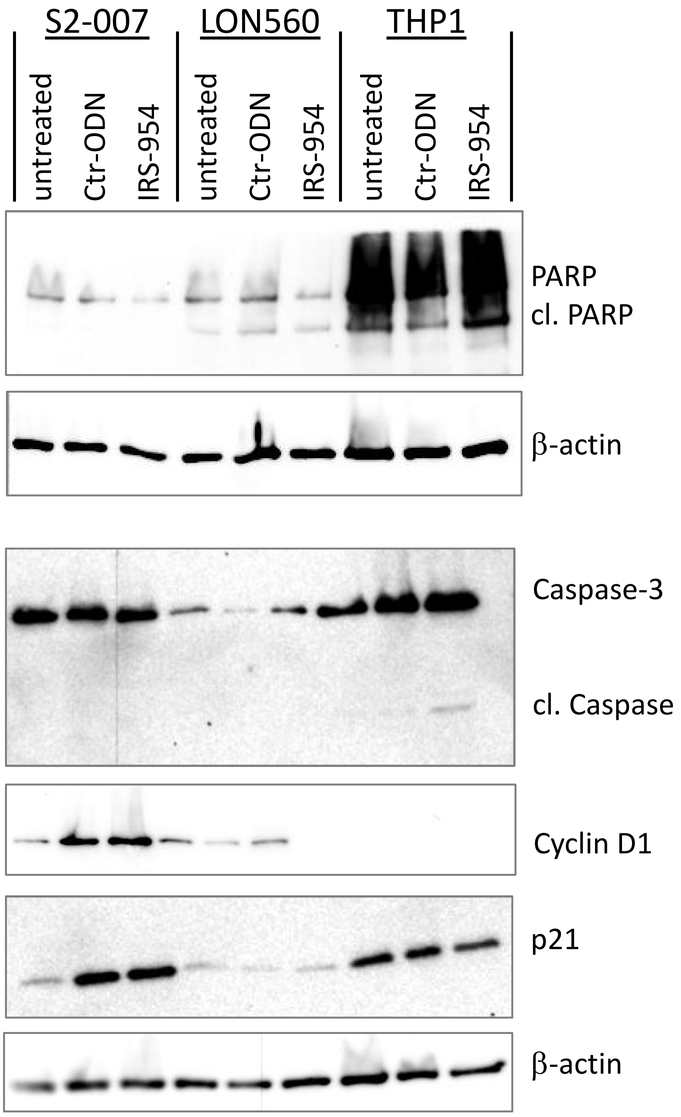

Supplementary Fig. 2: Inhibition of TLR7 by IRS-954 does not result in regulation of apoptosis or cell growth associated proteins

S2-007, LON560 and THP1 moncytic cells were treated with 10 µM IRS-954 or Ctr_ODN (n = 3-5). 72h after treatment cells were analyzed for levels of Caspase-3 and PARP or their cleavage, respectively, as well as for regulation of tumor suppressor p21 and growth controlling protein Cyclin D1 by western blot (n=3-5, exemplary data shown).
